# Antimicrobial resistance in *Neisseria gonorrhoeae* isolates at the Bangrak STIs Center, Bangkok, Thailand and WGS of antimicrobial-resistant isolates, 2018–23

**DOI:** 10.1093/jac/dkag260

**Published:** 2026-08-04

**Authors:** Porntip Paopang, Pongsathorn Sangprasert, Daniel Golparian, Natnaree Girdthep, Nutchaya Jintana, Supawadee Sonnak, Padchara Sangsuk, Thewarkrith Bunpan, Magnus Unemo, Rossaphorn Kittiyaowamarn

**Affiliations:** Bangrak STIs Center, Division of AIDS and STIs, Department of Disease Control, Thailand Ministry of Public Health, Nonthaburi, Thailand; Bangrak STIs Center, Division of AIDS and STIs, Department of Disease Control, Thailand Ministry of Public Health, Nonthaburi, Thailand; WHO Collaborating Centre for Gonorrhoea and Other STIs, Department of Laboratory Medicine, Faculty of Medicine and Health, Örebro University, Örebro, Sweden; Bangrak STIs Center, Division of AIDS and STIs, Department of Disease Control, Thailand Ministry of Public Health, Nonthaburi, Thailand; Bangrak STIs Center, Division of AIDS and STIs, Department of Disease Control, Thailand Ministry of Public Health, Nonthaburi, Thailand; Bangrak STIs Center, Division of AIDS and STIs, Department of Disease Control, Thailand Ministry of Public Health, Nonthaburi, Thailand; Department of Medical Technology and Clinical Pathology, Vachira Phuket Hospital, Phuket, Thailand; Department of Medical Technology and Clinical Pathology, Vachira Phuket Hospital, Phuket, Thailand; WHO Collaborating Centre for Gonorrhoea and Other STIs, Department of Laboratory Medicine, Faculty of Medicine and Health, Örebro University, Örebro, Sweden; Institute for Global Health, University College London, London, UK; Bangrak STIs Center, Division of AIDS and STIs, Department of Disease Control, Thailand Ministry of Public Health, Nonthaburi, Thailand

## Abstract

**Objectives:**

Antimicrobial resistance (AMR) in *Neisseria gonorrhoeae* is a global public health concern, particularly due to increasing resistance to the last remaining monotherapy, ceftriaxone, in several Asian countries. We examined the AMR of gonococcal isolates obtained at the Bangrak STIs Center, Bangkok, Thailand in 2018–23, and used WGS on isolates resistant to ceftriaxone, cefixime and/or azithromycin.

**Methods:**

Genital and extragenital swabs from males, females and transgender women were cultured. Etest (five antimicrobials) and EUCAST breakpoints (v16.0), or disc diffusion method (ciprofloxacin) were used to determine AMR. WGS was performed using Illumina NextSeq 550.

**Results:**

In 2018–23, 765 gonococcal isolates from 739 patients were cultured. Resistance to ceftriaxone, cefixime, azithromycin, tetracycline and ciprofloxacin was 0.5%, 0.8%, 1.7%, 83.7% and 95.7%, respectively. Ceftriaxone-resistant isolates, all resistant also to cefixime, were first detected in 2023 (*n* = 3, 1.2%). Additional cefixime-resistant isolates were found in 2022 (*n* = 2, 1.4%). Azithromycin-resistant isolates first emerged in 2020 (*n* = 1, 0.9%) and increased to nine (3.7%) isolates in 2023. All ceftriaxone-resistant isolates contained *penA*-60.001, but cefixime resistance was additionally caused by *penA*-34.019 and *penA*-10.001. All azithromycin-resistant isolates had 23S rRNA mutations and/or mosaic/semi-mosaic MtrD (subunit of the MtrCDE efflux pump).

**Conclusions:**

Resistance to ceftriaxone, cefixime and azithromycin has emerged in gonococcal strains circulating in Bangkok, Thailand, and resistance appears to be increasing. Resistance to these last options for monotherapy or dual therapy emphasize the need for strengthened national culture-based and genomic surveillance to prevent the spread of MDR and XDR strains in Thailand and globally, and implementation of the novel oral treatments, i.e. zoliflodacin and gepotidacin.

## Introduction

Gonorrhoea is a major global public health concern and, according to the WHO, in 2020 an estimated 82 million new cases of gonorrhoea occurred worldwide among adolescents and adults aged 15–49 years.^1^ In Thailand, the overall incidence of bacterial sexually transmitted infections (STIs), including gonorrhoea, non-gonococcal urethritis, syphilis, chancroid and lymphogranuloma venereum, has markedly increased in recent years, i.e. from 29.2 cases per 100 000 population in 2021 to 75.3 per 100 000 in 2024. Gonorrhoea has been the most common of these bacterial STIs, with the incidence increasing from 9.2 to 22.8 cases per 100 000 population in 2021–24.^2^

Antimicrobial resistance (AMR) in *Neisseria gonorrhoeae* has also become a global public health concern, compromising the treatment of gonorrhoea worldwide,^3,4^ and for several years WHO has listed *N. gonorrhoeae* as a high-priority pathogen for AMR research and surveillance.^5^ Emergence of MDR and XDR *N. gonorrhoeae* poses a serious threat to the effectiveness of the extended-spectrum cephalosporin (ESC) ceftriaxone, which is currently the only remaining option for empirical monotherapy of gonorrhoea.^3,4,6–16^ Notably, in some geographical settings the oral ESC cefixime and the macrolide azithromycin remain to be used, especially in empirical dual therapies.^3,6,7^ In 2015, the ceftriaxone-resistant FC428 clone, with the mosaic *penA*-60.001 allele (encoding a mosaic variant of the main ceftriaxone target, PBP2, which causes ceftriaxone resistance),^3,10–13,15,16^ was first identified in Japan,^17^ and since then its emerging *penA*-60.001-containing subtypes have been reported in many countries globally.^3,10–13,15,16,18–20^ Most confirmed ceftriaxone treatment failures worldwide have been linked to gonococcal strains originating in or being transmitted from Asia.^3,4,15,18,19^ In 2018, the first XDR *N. gonorrhoeae* strain with ceftriaxone resistance combined with high-level azithromycin resistance was isolated in the UK, but the infection was acquired in Thailand.^18^ Occasional ceftriaxone-resistant *N. gonorrhoeae* strains with links to Thailand have been subsequently reported in several European countries.^3,15,19^ In 2023, the first two ceftriaxone-resistant *N. gonorrhoeae* isolates, caused by *penA*-60.001, were detected in Thailand.^20^ In this emergent situation, the WHO Global Action Plan on AMR strongly stresses the need for sustained and high-quality AMR surveillance and research.^21^ However, continuous and quality-assured data on *N. gonorrhoeae* AMR in Asia remain limited as surveillance systems to monitor *N. gonorrhoeae* AMR are insufficient or absent in many Asian countries and in many other global settings.^3,4,21^ WGS has been shown to be highly valuable to supplement *N. gonorrhoeae* AMR surveillance locally, nationally and internationally, e.g. to elucidate the transmission of the pathogen and its AMR determinants.^11–13,22–24^

In the present study, we examined the antimicrobial susceptibility/resistance of 765 *N. gonorrhoeae* isolates obtained at the Bangrak STIs Center, Bangkok, Thailand, from January 2018 to December 2023, including the trends over the years. All *N. gonorrhoeae* isolates resistant to ceftriaxone, cefixime and/or azithromycin were also subject to WGS.

## Materials and methods

### Gonorrhoea patients and biosamples

The Bangrak STIs Center provides screening and treatment of STIs in the general population, including MSM and transgender women (TGW). From January 2018 to December 2023, individuals with suspected gonorrhoea (based on symptoms or risk behaviours) attending the Bangrak STIs Center, Bangkok, Thailand, were included. Clinical specimens were collected from multiple anatomical sites among the males, females and TGW. Briefly, urethral swabs were sampled from males (*n* = 4764), females (*n* = 4057) and TGW (*n* = 2). Endocervical samples were sampled from females (*n* = 4015), and vaginal and neovaginal swabs from 10 females and 5 TGW, respectively. Anorectal samples were collected from males (*n* = 1134) and females (*n* = 59), and oropharyngeal swabs from males (*n* = 2461), females (*n* = 2688) and TGW (*n* = 1). Swabs were sampled both for Gram-stained microscopy and culture (except for oropharyngeal swabs for which only culture was performed). Demographic data (sex and age) were collected for all patients with *N. gonorrhoeae*-positive culture. Notably, the Bangrak STIs Center is also involved in the WHO Enhanced Gonococcal Antimicrobial Surveillance Programme (EGASP);^25,26^ however, in the present study only gonorrhoea patients and gonococcal isolates from the routine diagnostics at the Bangrak STIs Center are reported and none of these patients or gonococcal isolates have been reported in the WHO EGASP, which only includes males with urethral discharge.

All patients were treated in accordance with the national Thai STI guidelines^27,28^ on their first visit, based on clinical evaluations and Gram-stained microscopy results. Consequently, based on detection of Gram-negative intracellular diplococci in microscopy and because non-gonococcal urethritis was not excluded, patients received ceftriaxone 250 mg (up to April 2019) or ceftriaxone 500 mg (from May 2019) plus azithromycin 1 g or doxycycline 100 mg two times per day for 7 days.^27–29^

### Laboratory diagnosis

One swab from urethra, endocervix, vagina, neovagina, anorectum and/or oropharynx was inoculated directly on selective gonococcal culture agar media (modified Thayer–Martin media) and the other swab from the same anatomical sites was used for Gram staining (except oropharyngeal swabs). Inoculated agar plates were incubated within a maximum of 30 min at 36 ± 1°C in a 5% CO_2_-enriched humid atmosphere for up to 48 h (examined daily). *N. gonorrhoeae* identification was performed using colony characteristics on selective gonococcal culture agar media, Gram-stained microscopy, oxidase test, superoxol test and a rapid micro carbohydrate test.^29^

### Antimicrobial susceptibility testing

MICs (mg/L) of ceftriaxone, cefixime, azithromycin, tetracycline and gentamicin were determined using the Etest (bioMérieux, Marcy-l'Étoile, France), in accordance with the manufacturer’s instructions. The disc diffusion method, in line with the US CLSI recommendations (www.clsi.org), was used for ciprofloxacin (ciprofloxacin 5 μg discs). *N. gonorrhoeae* ATCC 49226 and selected 2024 WHO reference strains (WHO K, L, P and/or WHO U)^30^ were included in each batch of antimicrobial susceptibility testing.

The following EUCAST clinical resistance breakpoints (www.eucast.org/clinical_breakpoints, v16.0) were used: ceftriaxone resistance MIC > 0.125 mg/L, cefixime resistance MIC > 0.125 mg/L, and tetracycline resistance MIC > 0.5 mg/L. The EUCAST epidemiological cut-off (ECOFF; MIC > 1 mg/L) was used to indicate resistance to azithromycin (www.eucast.org/clinical_breakpoints). For ciprofloxacin, a disc zone size of 27 mm indicated resistance (www.clsi.org). Notably, WHO recommends the disc diffusion method only for antimicrobial susceptibility testing of *N. gonorrhoeae* when MIC determination is not available or affordable.^4,30^ No resistance breakpoints for gentamicin have been stated by the EUCAST or US CLSI; accordingly, only the MICs of gentamicin are reported in the present study.

### WGS of isolates resistant to ceftriaxone, cefixime and/or azithromycin

DNA was extracted using the automated Qiasymphony DSP Virus/Pathogen kit (Qiagen, Hilden, Germany). Illumina DNA Prep (Illumina, Inc., San Diego, CA, USA) libraries were quality-controlled using Qubit (Thermo Fisher Scientific, Waltham, MA, USA) and Tapestation (Agilent Technologies, Santa Clara, CA, USA), according to the manufacturer’s instructions prior to library normalization. WGS using NextSeq 550 (Illumina, Inc.) and subsequent bioinformatic analysis were performed as previously described.^24^ Sequence reads are available from the European Nucleotide Archive (ENA, project accession PRJEB106070).

### Data analysis

A new gonococcal-positive culture more than 30 days after a previously treated gonorrhoea episode was considered as a new gonorrhoea episode in the same patient. Isolates from multiple anatomical sites in the same patient and visit with identical antimicrobial MIC profiles were considered as a single strain and only one of the isolates was included. Descriptive statistics (counts, proportions, etc.) were used to characterize the demographic data (sex and age) and for all *N. gonorrhoeae* isolates with available antimicrobial susceptibility data, both annually and cumulatively. MIC_50_, MIC_90_ and MIC ranges were determined. Data analysis was conducted using SPSS Version 23.0 (IBM Corp., Armonk, NY, USA).

## Results

### Gonorrhoea patient population

From 2018 to 2023, a total of 739 patients with *N. gonorrhoeae* infection (*n* = 765) were included (Table 1). Nineteen (2.6%) patients experienced more than one gonococcal infection, i.e. 18 (2.4%) patients experienced two episodes and 1 (0.1%) patient experienced three episodes of gonococcal infection. Furthermore, six (0.8%) patients were infected with two different strains at the same visit. Among the 739 patients, 408 (55.2%) were males and 331 (44.8%) were females, i.e. none of the TGW were *N. gonorrhoeae* culture-positive. Age was reported for 707 (95.7%) of patients, and the median age was 25 years for both the males and females (Table 1).

**Table 1.** Characteristics of gonorrhoea patients at the Bangrak STIs Center, Bangkok, Thailand, from January 2018 to December 2023

Characteristic	*n*	%
Age, y		
<15	12	1.6
15–19	122	16.5
20–24	202	27.3
25–29	156	21.1
30–34	85	11.5
35–39	44	6.0
40–44	38	5.1
≥45	48	6.5
Unreported	32	4.3
Sex		
Male	408	55.2
Female	331	44.8

### Antimicrobial susceptibility/resistance in Neisseria gonorrhoeae isolates (n = 765)

Resistance to ceftriaxone, cefixime, azithromycin, tetracycline and ciprofloxacin was 0.5%, 0.8%, 1.7%, 83.7% and 95.7%, respectively. The MICs of gentamicin ranged from 1 to 16 mg/L. All isolates resistant to ceftriaxone were collected in 2023 (*n* = 3, 1.2%), and five cefixime-resistant isolates were obtained in 2022 (*n* = 2, 1.4%) and 2023 (*n* = 3, 1.2%). No azithromycin-resistant isolates were identified before 2020, and all the 13 azithromycin-resistant isolates were collected in 2020 (*n* = 1, 0.9%), 2022 (*n* = 3, 2.1%) and 2023 (*n* = 9, 3.7%).

The minimum MIC, MIC_50_, MIC_90_ and maximum MIC values for ceftriaxone, cefixime, azithromycin, tetracycline and gentamicin by year are shown in Table 2. Briefly, ceftriaxone MICs ranged from ≤0.002 mg/L to 0.25 mg/L, and since 2018 the maximum MIC had increased from 0.032 mg/L to 0.25 mg/L. The MICs of cefixime varied from ≤0.016 mg/L to 1 mg/L; since 2018 the maximum MIC had increased from 0.125 mg/L to 1 mg/L. Finally, the azithromycin MICs ranged from ≤0.016 mg/L to >256 mg/L, and the maximum MIC had since 2018 increased from 1 mg/L to >256 mg/L (Table 2).

**Table 2.** MIC values for five antimicrobials in *Neisseria gonorrhoeae* isolates obtained at the Bangrak STIs Center, Bangkok, Thailand, from January 2018 to December 2023

Antimicrobial	MIC value, mg/L	2018	2019	2020	2021	2022	2023
Ceftriaxone	Tested isolates, *n*	45	70	78	83	145	245
	Min. MIC	≤0.002	≤0.002	≤0.002	≤0.002	≤0.002	≤0.002
	MIC_50_	0.004	0.004	0.004	0.004	0.004	0.004
	MIC_90_	0.008	0.008	0.008	0.016	0.016	0.016
	Max. MIC	0.032	0.064	0.064	0.032	0.125	0.25
Cefixime	Tested isolates, *n*	45	70	78	83	145	245
	Min. MIC	≤0.016	≤0.016	≤0.016	≤0.016	≤0.016	≤0.016
	MIC_50_	≤0.016	≤0.016	≤0.016	≤0.016	≤0.016	≤0.016
	MIC_90_	0.016	0.016	0.016	0.064	0.032	0.032
	Max. MIC	0.125	0.125	0.064	0.125	0.25	1
Azithromycin	Tested isolates, *n*	116	98	78	83	145	245
	Min. MIC	≤0.016	0.032	≤0.016	≤0.016	≤0.016	≤0.016
	MIC_50_	0.064	0.125	0.125	0.125	0.125	0.125
	MIC_90_	0.25	0.5	0.5	0.25	0.5	0.5
	Max. MIC	1	1	128	1	2	>256
Tetracycline	Tested isolates, *n*	116	98	78	83	145	245
	Min. MIC	0.25	0.25	0.25	0.5	0.125	0.125
	MIC_50_	16	16	16	16	8	16
	MIC_90_	16	32	16	16	16	16
	Max. MIC	64	64	32	32	32	64
Gentamicin	Tested isolates, *n*	116	98	78	83	145	245
	Min. MIC	2	4	4	2	1	2
	MIC_50_	4	8	8	8	4	8
	MIC_90_	8	8	8	8	8	8
	Max. MIC	16	8	16	16	16	16

Max., maximum; MIC_50_, MIC inhibiting 50% of isolates; MIC_90_, MIC inhibiting 90% of isolates; Min., minimum.

### WGS of isolates resistant to ceftriaxone, cefixime and/or azithromycin

All isolates with resistance to ceftriaxone, cefixime and/or azithromycin (*n* = 17) were successfully sequenced, and the corresponding gonorrhoea cases together with phenotypic and genomic characteristics of the isolates are summarized in Table 3. The ceftriaxone-resistant isolates were assigned to MLST ST8143 (*n* = 2) or ST16406 (*n* = 1), and *N. gonorrhoeae* sequence typing for antimicrobial resistance (NG-STAR) ST1771 (*n* = 2) or ST5793 (*n* = 1). The two additional cefixime-resistant isolates belonged to MLST (NG-STAR) ST11689 (ST199) and ST7363 (ST348). All the ceftriaxone-resistant isolates (*n* = 3) contained mosaic *penA*-60.001, and the cefixime resistance was caused by *penA*-60.001 (*n* = 3), *penA*-34.019 (*n* = 1) and *penA*-10.001 (*n* = 1). The azithromycin-resistant isolates (*n* = 13) belonged to eight MLST STs and eight NG-STAR STs. The resistance to azithromycin was caused by mosaic 2 of the MtrD subunit of the MtrCDE efflux pump (*n* = 4), 23S rRNA C2611T (*n* = 3), MtrD semi-mosaic 13 (*n* = 2), *mtrR* A-deletion (*n* = 2), 23S rRNA A2059G (*n* = 1), or 23S rRNA C2611T plus MtrD mosaic 2 (*n* = 1). One strain (No. 17) showed resistance to ceftriaxone plus high-level resistance to azithromycin, and this strain was assigned to MLST ST16406 and NG-STAR ST5793 (Table 3). Worryingly, this strain was obtained from a female sex worker. Nevertheless, all the three females with ceftriaxone-resistant gonococcal isolates were cured by recommended therapy (ceftriaxone 500 mg plus doxycycline 100 mg two times per day for 7 days; Table 3), i.e. all the females were *N. gonorrhoeae* culture-negative at test-of-cure.

**Table 3.** Characteristics of cases, including AMR determinants^a^ and molecular STs of *Neisseria gonorrhoeae* isolates (*n* = 17), with resistance to ceftriaxone, cefixime and/or azithromycin at the Bangrak STIs Center, Bangkok, Thailand, January 2018 to December 2023

Year detected	Age	Sex	Sex partner	Anatomical site of isolate	Treatment received	MIC values (mg/L; Etest^b^)			*penA* allele	23S rRNA	MtrD	*mtrR* A-deletion	MLST	NG-STAR CC
						CRO	CFM	AZM						
2020	32	M	Not reported	REC	Not reported	0.008	0.016	**128**	2.001	C2611T	Mosaic 2	WT	9363^a^	213^a^
2022	27	M	M	REC	CRO 500 mg + AZM 1 g + DOX^c^	0.064	**0**.**25**	1	34.019	WT	Semi-mosaic 13	A-deletion	11689	199
2022	30	F	M	CX	CRO 500 mg + DOX^c^	0.008	0.016	**2**	2.002	C2611T	Non-mosaic	WT	1587	719
2022	26	F	M	PH	CRO 500 mg + DOX^c^	0.008	0.016	**2**	2.002	C2611T	Non-mosaic	WT	1587	719
2022	28	M	Not reported	URE	Not reported	0.016	0.016	**2**	2.001	WT	Mosaic 2	WT	11422^a^	168^a^
2022	37	F	M	CX	CRO 500 mg + DOX^c^	0.125	**0**.**25**	0.5	10.001	WT	Non-mosaic	WT	7363	348
2023	22	F	M	CX	CRO 500 mg + DOX^c^	**0**.**25**	**1**	0.5	60.001	WT	Non-mosaic	WT	8143	1771
2023	20	F	Not reported	Eye	Not reported	0.008	0.016	**2**	2.001	WT	Mosaic 2	WT	9363	168
2023	27	F	M	CX	CRO 500 mg + DOX^c^	0.002	0.016	**32**	5.002	C2611T	Non-mosaic	WT	11706	1615
2023	22	F	M	CX	CRO 500 mg + DOX^c^	**0**.**25**	**1**	0.25	60.001	WT	Non-mosaic	WT	8143	1771
2023	33	M	M	REC	CRO 500 mg + DOX^c^	0.008	0.016	**2**	2.001	WT	Non-mosaic	A-deletion	13527	166
2023	19	M	M	REC	CRO 500 mg + DOX^c^	0.008	0.016	**2**	2.001	WT	Non-mosaic	A-deletion	9362	166
2023	22	M	M	REC	CRO 500 mg + DOX^c^	0.004	0.016	**2**	2.001	WT	Semi-mosaic 13	A-deletion	17948	166
2023	33	M	M	URE	CRO 500 mg + DOX^c^	0.004	0.016	**2**	2.001	WT	Semi-mosaic 13	A-deletion	17948	436
2023	26	M	Not reported	URE	Not reported	0.008	0.016	**2**	2.001	WT	Mosaic 2	WT	11422^a^	168^a^
2023	21	F	M	CX	CRO 500 mg + DOX^c^	0.008	0.016	**2**	2.001	WT	Mosaic 2	WT	9363	1489
2023	29	F	M	CX	CRO 500 mg + DOX^c^	**0**.**25**	**1**	**>256**	60.001	A2059G	Non-mosaic	WT	16406	5793

AZM, azithromycin; CRO, ceftriaxone; CFM, cefixime; CX, endocervix; DOX, doxycycline; F, female; M, male; NG-STAR, *N. gonorrhoeae* sequence typing for antimicrobial resistance; PH, pharyngeal; REC, rectal; URE, urethra.

^a^No known resistance determinant for the novel antimicrobials zoliflodacin (in GyrB D429 or K450)^31–33^ or gepotidacin (in GyrA A92)^34–36^ was found. However, the ParC D86N^34–37^ mutation that can predispose for resistance to gepotidacin was found in 47.1% (8/17) of isolates. Furthermore, with exception of three isolates [MLST ST11422, NG-STAR CC168 (*n* = 2) and MLST ST9363, NG-STAR CC213 (*n* = 1)], all isolates contained a GyrA S91F substitution, which confirmed their resistance to ciprofloxacin.^22–24^

^b^MIC values representing resistance according to the current EUCAST breakpoints (www.eucast.org/clinical_breakpoints) are in bold letters.

^c^Doxycycline 100 mg two times per day for 7 days.^27–29^

## Discussion

The present study describes the antimicrobial susceptibility/resistance in *N. gonorrhoeae* isolates obtained at the Bangrak STIs Center, Bangkok, Thailand, from January 2018 to December 2023. Among the 765 *N. gonorrhoeae* isolates obtained from 739 patients, resistance to ceftriaxone, cefixime and azithromycin was 0.5%, 0.8% and 1.7%, respectively, while high resistance rates were observed for tetracycline (83.7%) and ciprofloxacin (95.7%). All ceftriaxone-resistant *N. gonorrhoeae* isolates (which were also resistant to cefixime) were detected in 2023, and azithromycin-resistant isolates were observed since 2020. Data from the WHO EGASP in Thailand from 2015 to 2021 showed that all *N. gonorrhoeae* isolates remained susceptible to ceftriaxone, and only 0.26% of isolates were resistant to cefixime (all detected in 2020 and 2021).^26^ Furthermore, no ceftriaxone-resistant isolates were detected in Thailand in the WHO EGASP 2023 data either.^25^ However, in 2023 the first two ceftriaxone-resistant *N. gonorrhoeae* isolates, caused by *penA*-60.001, were detected in the national surveillance in Bangkok, Thailand.^20^ The current study detected ceftriaxone-resistant and cefixime-resistant isolates collected in 2022 and 2023, indicating a continued emergence and spread of resistance to these ESCs in Thailand. Thus, international GASPs, such as the WHO EGASP,^10–13,25,26^ are exceedingly valuable. However, they capture only a limited number of gonorrhoea cases in each country and, most frequently, they only include urethral samples from symptomatic males with urethritis and/or urethral discharge, because of cost-efficiency due to the high gonococcal-positivity rate in culture. Consequently, national and local gonococcal AMR surveillance, in GASPs or routine diagnostics, that ideally also culture samples from females and extragenital sites (oropharynx and anorectum) are important to supplement the international initiatives where feasible and affordable. For example, in the present study 9 of the 17 cases with isolates resistant to ceftriaxone, cefixime and/or azithromycin were female cases, and among the remaining 8 cases (in males), 5 were anorectal infections. Accordingly, culturing only male urethral samples would have missed 14 of these 17 cases [including all the ceftriaxone-resistant isolates (*n* = 3) obtained from females]. Notably, the ceftriaxone and cefixime resistance levels detected in Bangkok, Thailand, remain substantially lower than in Asian countries such as Cambodia,^10,11,25,38^ China^14–16^ and Vietnam.^12,13,25,38^ This is despite the relatively comprehensive WHO EGASP (active in several sentinel sites in Thailand since 2015)^25,26^ and national gonococcal AMR surveillance in Thailand (active since 2003).^39^ It cannot be excluded that the emergence of ceftriaxone resistance in Thailand remains in an early stage and/or it is concentrated in some hard-to-reach populations (e.g. some groups of sex workers) not captured in the culture-based WHO EGASP and national surveillance. Notably, in January–April 2025, five gonorrhoea cases caused by ceftriaxone-resistant *penA*-60.001-harbouring gonococcal strains were confirmed in the national gonococcal AMR surveillance in Thailand.^39^ The frequent practice of self-treatment by purchase of antimicrobials without prescription at pharmacies in Thailand,^40^ associated with inappropriate and failing treatment and further driving of AMR, is a major concern. Consequently, enhanced gonococcal AMR surveillance in all risk groups and populations in conjunction with WGS and patient-level metadata would be most valuable in Thailand. Anyway, it is a major concern that ceftriaxone-resistant strains, including with high-level azithromycin resistance, spreading internationally, such as *penA*-60.001-containing MLST ST16406 and NG-STAR ST5793 strains,^11–13,15,16,38^ have also been detected in Thailand since 2023. In Thailand and other countries where ceftriaxone resistance is detected, it is imperative to promptly implement the WHO recommended 1 g dose of ceftriaxone as first-line treatment of gonorrhoea, which is also in line with the updated gonorrhoea management guidelines in Thailand.^41^ Ceftriaxone 1 g has been shown *in vitro* and *in vivo* to cure the vast majority of currently detected cases of gonorrhoea caused by ceftriaxone-resistant strains, which mostly have a ceftriaxone MIC of 0.25–0.5 mg/L.^15,19,42^ However, oropharyngeal gonococcal infections and infections caused by gonococcal strains with ceftriaxone MICs >0.5 mg/L are more likely to fail cure with ceftriaxone 1 g treatment.^15,19,42^ Notably, all the ceftriaxone-resistant strains appear susceptible to the novel antimicrobials zoliflodacin^31–33,37^ and gepotidacin,^34–36,43,44^ and it is essential that these antimicrobials are licensed in a timely manner and available for treatment of uncomplicated gonorrhoea in Thailand and many other countries.

In conclusion, resistance to ceftriaxone, cefixime and azithromycin in gonococcal isolates obtained at the Bangrak STIs Center, Bangkok, Thailand, has emerged and appears to be increasing. Although AMR levels are lower than in Asian countries such as Cambodia,^10,11^ China^14,16^ and Vietnam,^12,13^ the emergence of these AMR strains in Thailand strongly emphasizes the need for strengthened national culture-based and genomic surveillance in conjunction with implementation of ceftriaxone 1 g for first-line gonorrhoea therapy and the novel therapeutic oral antimicrobials zoliflodacin^31–33,37^ and gepotidacin,^34–36,43,44^ which will be essential for maintaining effective prevention and treatment of MDR and XDR *N. gonorrhoeae* in Thailand. Finally, the frequent practice of self-treatment by purchase of antimicrobials without prescription at pharmacies in Thailand,^40^ often resulting in inappropriate and failing treatment and further driving of AMR, is a major concern. Accordingly, enhanced responsible antimicrobial use and antimicrobial stewardship to protect both individual and public health is also of utmost importance.
